# Impacts of COVID-19 on Nutritional Intake in Rural China: Panel Data Evidence

**DOI:** 10.3390/nu14132704

**Published:** 2022-06-29

**Authors:** Xinru Han, Yufei Guo, Ping Xue, Xiudong Wang, Wenbo Zhu

**Affiliations:** 1 Institute of Agricultural Economics and Development, Chinese Academy of Agricultural Sciences, Beijing 100081, China; hanxinru@caas.cn (X.H.); 82101201276@caas.cn (P.X.); wangxiudong@caas.cn (X.W.); 2 School of Advanced Agricultural Sciences, Peking University, Beijing 100871, China; 2018311179@email.cufe.edu.cn; 3 School of Economics, Central University of Finance and Economics, Beijing 102206, China; 4 Rural Development Institute, Chinese Academy of Social Sciences, Beijing 100732, China

**Keywords:** COVID-19, nutritional intake, rural China, food consumption, food security

## Abstract

The COVID-19 pandemic introduced risks and challenges to global food and nutrition security. In this paper, we examine the impact of the COVID-19 pandemic on the nutritional intake of China’s rural residents using panel data and a fixed effects model. The data were collected in 2019 and 2020 and covered nine provinces and 2631 households in rural China. The results reveal that an increase of 100 confirmed cases in a county resulted in a 1.30% (*p* < 0.01), 1.42% (*p* < 0.01), 1.65% (*p* < 0.01), and 1.15% (*p* < 0.01) decrease in per capita intake of dietary energy, carbohydrates, fats, and proteins, respectively. Moreover, the COVID-19 pandemic had a significant and negative effect on dietary macronutrient intake in the low-income group at the 5% level of significance. Our study indicates that the potential insufficient nutrition situation, nutritional imbalance, and dietary imbalance of low-income rural residents should be addressed appropriately.

## 1. Introduction

The COVID-19 pandemic has been ongoing since January 2020 as a result of its rapid and widespread transmission and its difficulty in prevention and control [1,2,3]. As of 13 May 2022, there were 517,648,631 confirmed cases, including 6,261,708 deaths worldwide [4]. The epidemic has had a profound impact on the global economy and welfare, such as business shutdowns, job losses, disrupted supply chains, commodity price volatility, etc. [5,6,7,8]. Moreover, the pandemic introduced risks and challenges to global food and nutrition security [9,10] and made the pathway towards SDG2 even steeper [11], especially in rural areas of the developing world [12,13,14,15]. The channels through which the pandemic affects food and nutrition security comprise all four pillars of food security. Food availability and stability are affected by a lack of workers [16], delays in agricultural work [17], an increase in the price of food and materials [18,19,20,21], and trade restrictions [22]. Moreover, major threats to food access and utilization posed by COVID-19 are the loss of household income, reduced purchasing power [23,24,25], and supply chain disruptions caused by lockdown measures [26,27,28].

In this paper, we examine the impact of the COVID-19 pandemic on nutritional intake, a key aspect of food utilization and SDG2 [29]. Recent literature shows that the pandemic has had a significant but heterogeneous impact on nutritional intake. The consumption of nutrient-dense foods, such as vegetables, fruit, and animal-source food, has been reduced, while the consumption of carbohydrate-containing foods, such as bread, increases [30,31,32]. However, the lockdown policy led to an increase in fruit, vegetables, and fat consumption in some developed countries [33]. In terms of specific populations, the pandemic impact on Dutch older adults was negative [34], while the impact on Australian university students was positive [35]. Furthermore, evidence shows that the COVID-19 pandemic might affect the dietary structure and consumer behavior [36,37]. For example, consumers may prefer healthy diets [38,39], the demand for online food delivery may increase [40,41], panic buying may occur [42], and sustainable food consumption may be promoted [43,44,45].

The COVID-19 pandemic seriously affected rural China [32]. About 27% of the agri-food system’s workers (about 46 million) lost their jobs due to COVID-19 during the lockdown phase (January 2020–March 2020) [46]. According to a survey in mid-February 2020, 23% of households who have been out of poverty since 2013 believed they might return to poverty [47]. However, only a few studies have evaluated the impacts of the COVID-19 pandemic on the dietary diversity [48,49] and food consumption of China’s rural residents [50]. Tian et al. (2022) found that COVID-19 positively affected rural households’ consumption of vegetables, aquaculture, and legumes, but COVID-19 significantly reduced rural households’ dietary diversity [50]. To the best of our knowledge, the pandemic’s impact on the nutritional intake of China’s rural residents is still unknown.

To fill the research gaps and enable a better understanding of how the COVID-19 pandemic affects nutritional intake, the specific objectives of the study are to: (i) investigate the COVID-19 pandemic impact on the nutritional intake of China’s rural residents; and (ii) identify the heterogeneity of the pandemic impact among different income groups in addition to considering the different impacts of the pandemic on countries with different income levels.

Moreover, since most similar studies use cross-sectional data [34,35] or non-national and small-size panel data [50], we use a nationwide panel data with nine provinces and 2,631 rural households and a fixed effects model following Amare et al. (2021) [23] to control for the unobserved factors, such as dietary preferences. Given China’s food security concerns in the future [51,52], this study can provide policy recommendations for securing the basic application needs of rural residents.

## 2. Materials and Methods

### 2.1. Study Design

We empirically evaluated the impact of the COVID-19 pandemic on Chinese rural residents’ nutritional intake using a multiple fixed effects (FE) model. The baseline regression is as follows.

(1) $$\mathrm{Ln}Nutrition_{hcpt}=\beta_{0}+\beta_{1}COVID_{ct}+\beta X_{hcpt}+\alpha_{h}+\varepsilon_{hcpt}$$

where the outcome variable $Nutrition_{hcpt}$ indicates the quantity of the nutritional intake of household *h* in county *c*, province *p*, and time *t*. In this paper, the outcome variable includes dietary energy, carbohydrate, fat, and protein. $COVID_{ct}$ is the key explanatory variable, indicating the number of confirmed COVID-19 cases. $X_{hcpt}$ is a matrix of control variables, including the price of nutrients, expenditure, number of days spent performing non-farm work, presence of heavy workers, sports facilities in villages, total retail sales of consumer goods in counties, Internet access, and family size. $\alpha_{h}$ is the household fixed effect, and $\varepsilon_{hcpt}$ is the error term. $\beta_{1}$ is the key parameter indicating the impact of COVID-19 on nutritional intake, indicating one more confirmed case in a county would result in a 100 × $\beta_{1}$% change in nutrient intake in ceteris paribus condition.

To control for the unobservable aspects that stay constant within the county, province, and time, we add three more parameters to Equation (1).

(2) $$\mathrm{Ln}Nutrition_{hcpt}=\beta_{0}+\beta_{1}COVID_{ct}+\beta X_{hcpt}+\alpha_{h}+\delta_{c}+\theta_{p}+\gamma_{t}+\varepsilon_{hcpt}$$

where $\delta_{c}$ is county fixed effect, which controls all time-invariant county-level characteristics. Moreover, $\theta_{p}$ and $\gamma_{t}$ indicate the province and time fixed effect, respectively.

### 2.2. Data Collection

**Figure 1:**
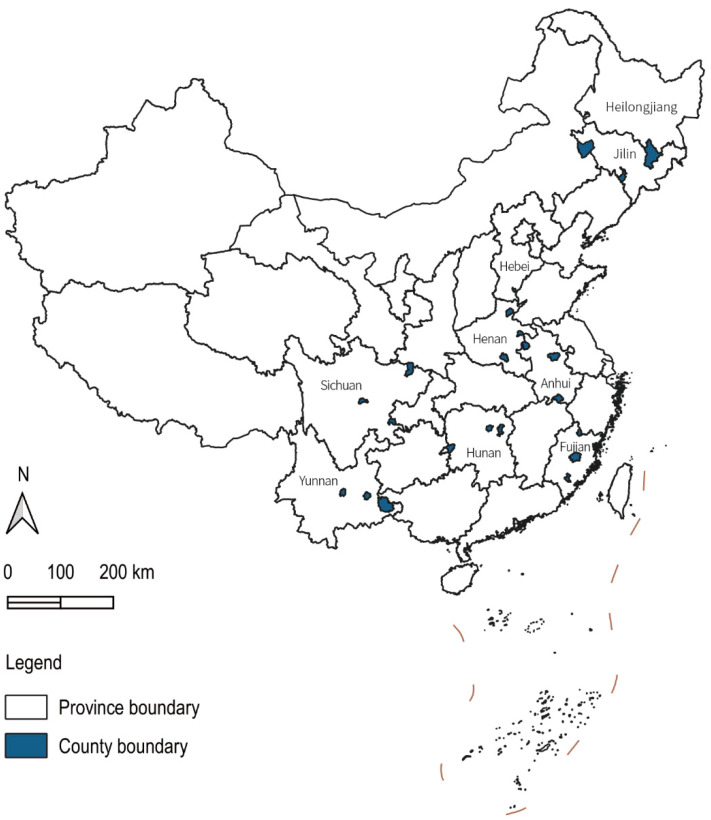
The geographical location of the study areas.

We used the 2019–2020 Survey for Agriculture and Village Economy (SAVE) data collected by the Institute of Agricultural Economics and Development, Chinese Academy of Agricultural Sciences [53,54,55]. The 2019–2020 SAVE data record the annual production, consumption, expenditure, and income of the rural households and cover 5818 observations in the Hebei, Jilin, Heilongjiang, Anhui, Fujian, Henan, Hunan, Sichuan, and Yunnan provinces of China (Figure 1). Moreover, the number of accumulated confirmed COVID-19 cases in each county by the end of December 2020 was collected by Wind Info. We also used the consumer price index (CPI) data from the National Bureau of Statistics of China (NBSC).

### 2.3. Outcome Variables

Since the SAVE data only contains at-home consumption information of households for 18 food items, we first divided the household food consumption by the family size to obtain the per capita food consumption (kg/year), then converted the per capita food consumption into per capita intake of dietary energy (kcal/day), carbohydrates (g/day), fat (g/day), and protein (g/day), based on the China Food Composition [56].

However, this method may have underestimated the nutritional intake because it ignores other food (not included in the 18 categories) consumed at home and all food consumed away from home. We assumed that the nutritional content of other food consumed at home and all food consumed away from home was proportional to the 18 categories of food consumed at home as a function of expenditure [57]. Meanwhile, we assumed 50% of food expenditures away from home pertained to food quantities consumed [58]. Thus, the proportion of the 18 categories of food expenditure in the total food expenditure can be expressed as follows:

(3) $$\omega =\underset{i}{\sum }x_{i}/\left(\underset{i}{\sum }x_{i}+X_{OT}+0.5X_{FAFH}\right)$$

where *I* = 1,…,18; $x_{i}$ represents the expenditure on food item *i*; $X_{OT}$ indicates the expenditure on other food (not included in the 18 categories) consumed at home; $X_{FAFH}$ indicates the food expenditure away from home. Thus, the per capita daily intake of nutrient *k* is expressed as:

(4) $$Nutrition_{k}=\sum_{i}N_{ik}q_{i}\gamma_{i}/\omega$$

where $Nutrition_{k}$ represents the total intake of nutrient *k* from all food items (Table 1); $N_{ik}$ is the intake of nutrient *k* obtained from food item *i*; $q_{i}$ represents the per capita consumption of food item *i*; and $\gamma_{i}$ represents the proportion of the edible parts of food item *i*.

### 2.4. Control Variables

#### 2.4.1. COVID-19

According to the *Law on the Prevention and Control of Infectious Diseases of the People’s Republic of China*, the county government can take measures such as stopping work, restricting activities, or lockdown as necessary for public safety. Further, there have been differences in prevention and control policies among counties in China during the COVID-19 pandemic. Thus, we use the cumulative cases at the county level to measure the impact of COVID-19 (Table 1).

#### 2.4.2. Weighted Price of Nutrients

Price is one of the major determinants of consumer behavior [59,60,61]. As a consequence of the lockdown policies implemented by COVID-19, the food purchase and nutrition intake of rural residents were strongly influenced by price fluctuations [18,62]. However, it was only possible to collect food prices (unit values), not nutrient prices, during the data collection process. Thus, a weighted nutrition price ($P_{k}$) is introduced in this paper to describe the price of nutrients.

(5) $$P_{k}=\sum_{i=1}^{18}\left(\frac{N_{ik}}{Nutrition_{k}}\times \frac{P_{i}}{N_{ik}}\right)=\sum_{i=1}^{18}\left(\frac{N_{ik}}{Nutrition_{k}}\times \frac{E_{i}/Q_{i}}{N_{ik}}\right)$$

where $P_{i}$ is the price of food item *i* (Table 1); $E_{i}$ and $Q_{i}$ indicate the expenditure and consumed quantity of food item *i*, respectively. Further, $\frac{P_{i}}{N_{ik}}$ indicates the price (or the unit values) of nutrient *k* in food item *i*, and $\frac{N_{ik}}{Nutrition_{k}}$ indicates the proportion of nutrient *k* obtained from food item *i* in the total intake of nutrient *k* from all food items.

#### 2.4.3. Other Control Variables

Income, expenditure, and family size are also important determinants of food consumption (Table 1) [59,63,64,65]. In the single equation model of food consumption, either income or expenditure can be used. In this paper, the per capita annual expenditure was used since respondents usually do not provide their actual incomes. We also used an instrumental estimation of the fixed effect model and used expenditure as the instrumental variable of income. Moreover, activity level and food accessibility are also variables that could affect nutritional intake [26,41]. Though the variables are not available in the SAVE data, we selected some proxy variables (Table 1). As proxies for activity level, we utilized the existence of sports facilities in villages, the number of days spent performing non-farm work by household laborers, and the presence of heavy workers in the industry, construction, and mining. As proxies for food accessibility, we chose the total retail sales of consumer goods in counties and whether households had access to the Internet.

**Table 1:** Definitions of major variables.

Variable	Definition	Unit
*COVID*	Cumulative cases in the county by the end of 2020	Hundred cases
*Carbohydrate*	Per capita carbohydrate intake	g/day
*Fat*	Per capita fat intake	g/day
*Protein*	Per capita protein intake	g/day
*Energy*	Per capita dietary energy intake	kcal/day
*Price_ch*	Weighted price of carbohydrates	CNY/kg
*Price_fat*	Weighted price of fat	CNY/kg
*Price_pt*	Weighted price of protein	CNY/kg
*Price_energy*	Weighted price of dietary energy	CNY/1000 kcal
*Inc*	Per capita annual income	1000 CNY
*Exp*	Per capita annual expenditure	1000 CNY
*Family size*	Number of family members	/
*Non-farm work*	Number of days spent performing non-farm work by household laborers	days
*Heavy work*	Presence of heavy workers in the industry, construction, and mining	Yes = 1, No = 0
*Sport*	Existence of sports facilities in villages	Yes = 1, No = 0
*Retail*	Total retail sales of consumer goods in counties	100 million CNY
*Internet*	Whether households had access to the Internet	Yes = 1, No = 0
*Year2020*	=1 (year = 2020); =0 (year = 2019)	/

### 2.5. Data Processing and Cleaning

First, we deleted some samples to construct balanced panel data. Second, we excluded samples with extreme values by winsorizing at the 2% quantile. Third, prices, incomes, and expenditures were deflated by China’s annual CPI. After data processing and cleaning, we kept 2631 rural households, and the total observation was 5262 (Figure 1).

### 2.6. Statistical Analysis

As shown in Table 2, the average per capita daily intakes of carbohydrates, fat, protein, and dietary energy in 2019 were 252.88 g, 96.72 g, 48.56 g, and 2059.43 kcal, respectively. In 2020, the average carbohydrate intake decreased by 5.43 g, while fat and protein intakes increased by 2.01 g and 0.17 g, respectively. However, the differences in macronutrient intakes were not significant. In terms of data quality, the per capita daily intake of dietary energy was similar to the *Report on the Nutrition and Chronic Disease Status of Chinese Residents* (2020) [66]. However, the fat intake from the SAVE data was higher than that of the *Report on the Nutrition and Chronic Disease Status of Chinese Residents* (2020), while the carbohydrate and carbohydrate intakes from the SAVE data were lower.

The average unit price of macronutrients has increased from 2019 to 2020. The average unit price of fat, protein, and dietary energy significantly increased in 2020, by 0.48 CNY, 0.98 CNY, and 0.03 CNY, respectively, whereas the difference in the average unit price of carbohydrates did not change significantly. Additionally, while per capita income in 2020 was essentially the same as it was in 2019, per capita expenditure was significantly higher.

Compared with 2019, the proxy variables for the activity level in 2020 were stable. There were an average of 3.94 family members, 105 days of non-farm work were spent per year, 30% of household laborers engaged in heavy work including industry, construction, and extraction, and sports facilities were found in about 53% of villages. On the other hand, overall retail sales of consumer goods in counties decreased significantly by approximately 741 million CNY compared with 2019 due to embargo restrictions and the closure of some retail businesses. Meanwhile, a significant increase of 10 percentage points has been observed in the proportion of farmers with Internet access.

**Table 2:** Summary statistics for major variables.

Variable	Full Sample		Pre-COVID-19 (2019)		Post-COVID-19 (2020)		Diff. in Means (2020–2019)
	Mean	SD	Mean	SD	Mean	SD	
*COVID*	0.42	0.97	0.00	0.00	0.85	1.23	0.85 ***
*Carbohydrate*	250.17	138.82	252.88	140.21	247.45	137.39	−5.43
*Fat*	97.72	45.31	96.72	44.47	98.73	46.13	2.01
*Protein*	48.65	22.91	48.56	22.58	48.73	23.24	0.17
*Dietary_Energy*	2058.25	908.62	2059.43	901.08	2057.08	916.28	−2.34
*Price_ch*	6.26	11.42	6.06	10.88	6.47	11.94	0.41
*Price_fat*	10.83	8.54	10.59	7.85	11.07	9.17	0.48 **
*Price_pt*	18.59	7.91	18.10	7.19	19.08	8.56	0.98 ***
*Price_energy*	0.49	0.22	0.48	0.21	0.51	0.23	0.03 ***
*Inc*	17.96	19.15	17.99	18.76	17.93	19.53	−0.06
*Exp*	36.38	17.64	35.86	17.35	36.90	17.92	1.04 **
*Family size*	3.94	1.59	3.93	1.60	3.95	1.59	0.02
*Non-farm work*	105.07	102.26	104.82	101.53	105.32	103.01	0.50
*Heavy work*	0.30	0.46	0.30	0.46	0.31	0.46	0.01
*Sport*	0.53	0.50	0.53	0.50	0.53	0.50	0.00
*Retail*	100.64	114.35	104.35	117.82	96.94	110.66	−7.41 **
*Internet*	0.67	0.47	0.62	0.49	0.72	0.45	0.10 ***
Observations	5262		2631		2631		

Notes: ** *p* < 0.05, *** *p* < 0.01.

## 3. Results

In this section, we first presented the estimation results of the COVID-19 impact on dietary energy, carbohydrate, fat, and protein intakes, respectively. Then, we proved the robustness of the estimation results. Finally, we identified the heterogeneity in pandemic impact across different income groups.

### 3.1. COVID-19 Impact on Dietary Energy Intake

Table 3 sheds light on the impacts of COVID-19 on dietary energy intake. To explore the nonlinear relationship between dietary energy intake and expenditure, we added the square term of the expenditure into Equation (2). As shown in Table 3, a negative and significant coefficient of *COVID* indicates that an increase in COVID-19 cases in the counties will significantly reduce the per capita dietary energy intake of rural residents. Specifically, an increase of 100 confirmed cases in a county results in a 1.30% (*p* < 0.01) decrease in per capita dietary energy intake (Table 3).

In addition, our results demonstrate that an increase in weighted energy price led to a decrease in dietary energy intake. The dietary energy intake will decrease by approximately 0.48% (*p* < 0.01) for every 1% increase in price (Table 3). Accordingly, the coefficient on the square term of the expenditure was significantly negative, which indicates that the impact of expenditure on dietary energy intake had an inverted U-shape. Furthermore, the results indicate that a larger family tended to reduce the dietary energy intake of family members, in line with previous research.

**Table 3:** Estimation results of the COVID-19 impact on dietary energy intake.

Variables	FE Model 1 (1)	FE Model 2 (2)	FE Model 3 (3)	FE Model 4 (4)
*COVID*	−0.0130 ***	−0.0130 ***	−0.0130 ***	−0.0130 ***
	(0.0036)	(0.0036)	(0.0036)	(0.0036)
ln*Price_energy*	−0.48 ***	−0.48 ***	−0.48 ***	−0.48 ***
	(0.02)	(0.06)	(0.06)	(0.06)
ln*Exp*	2.40 ***	2.40 ***	2.40 ***	2.40 ***
	(0.57)	(0.83)	(0.83)	(0.83)
(ln*Exp*)^2^	−0.11 ***	−0.11 ***	−0.11 ***	−0.11 ***
	(0.03)	(0.04)	(0.04)	(0.04)
*Family size*	−0.04 ***	−0.04 **	−0.04 **	−0.04 **
	(0.01)	(0.02)	(0.02)	(0.02)
*Non-farm work*	−0.00	−0.00	−0.00	−0.00
	(0.00)	(0.00)	(0.00)	(0.00)
*Heavy work*	−0.01	−0.01	−0.01	−0.01
	(0.01)	(0.02)	(0.02)	(0.02)
*Sport*	0.04 **	0.04	0.04	0.04
	(0.01)	(0.02)	(0.02)	(0.02)
ln*Retail*	0.05 *	0.05	0.05	0.05
	(0.03)	(0.05)	(0.05)	(0.05)
*Internet*	−0.01	−0.01	−0.01	−0.01
	(0.01)	(0.02)	(0.02)	(0.02)
Constant term	−6.30 **	−6.30	−6.30	−6.30
	(2.96)	(4.26)	(4.27)	(4.27)
Year FE	YES	YES	YES	YES
Household FE	YES	YES	YES	YES
County FE	NO	NO	YES	YES
Province FE	NO	NO	NO	YES
Cluster robust standard errors	None	Village	Village	Village
Observations	5262	5262	5262	5262

Notes: Standard errors in parentheses; * *p* < 0.10, ** *p* < 0.05, *** *p* < 0.01.

### 3.2. COVID-19 Impact on Carbohydrate, Fat, and Protein Intakes

From Table 4, Table 5 and Table 6, the most important highlight is that the increased number of confirmed COVID-19 cases in a county caused a significant reduction in per capita carbohydrate, fat, and protein intake. For every 100 additional cases of COVID-19 in a county, the intake of carbohydrates, fats, and proteins declined by 1.42% (*p* < 0.01), 1.65% (*p* < 0.01), and 0.81% (*p* < 0.01), respectively. Thus, among the three major macronutrients, COVID-19 had the largest relative effect on fat intake in rural China.

In addition, the own-price elasticities of the three macronutrients were negative, and the cross-price elasticities were positive (Table 4, Table 5 and Table 6). The own-price elasticities of carbohydrate, fat, and protein were −0.87 (*p* < 0.01), −0.76 (*p* < 0.01), and −0.69 (*p* < 0.01), respectively. The result indicates that Chinese rural residents were most sensitive to the price of carbohydrates, and the macronutrients had a significant substitution relationship. An increase in the price of one nutrient will result in the consumer switching to another nutrient to ensure adequate overall calorie intake.

**Table 4:** Estimation results of the COVID-19 impact on carbohydrate intake.

Variables	FE Model 1 (1)	FE Model 2 (2)	FE Model 3 (3)	FE Model 4 (4)
*COVID*	−0.0142 ***	−0.0142 ***	−0.0142 ***	−0.0142 ***
	(0.0038)	(0.0035)	(0.0035)	(0.0035)
*lnPrice_ch*	−0.87 ***	−0.87 ***	−0.87 ***	−0.87 ***
	(0.02)	(0.05)	(0.05)	(0.05)
*lnPrice_fat*	0.32 ***	0.32 ***	0.32 ***	0.32 ***
	(0.02)	(0.06)	(0.06)	(0.06)
*lnPrice_pt*	0.16 ***	0.16 *	0.16 *	0.16 *
	(0.04)	(0.09)	(0.09)	(0.09)
ln*Exp*	2.53 ***	2.53 ***	2.53 ***	2.53 ***
	(0.60)	(0.92)	(0.92)	(0.92)
(ln*Exp*)^2^	−0.11 ***	−0.11 **	−0.11 **	−0.11 **
	(0.03)	(0.04)	(0.04)	(0.04)
*Family size*	−0.04 ***	−0.04 **	−0.04 **	−0.04 **
	(0.01)	(0.02)	(0.02)	(0.02)
*Non-farm work*	−0.00	−0.00	−0.00	−0.00
	(0.00)	(0.00)	(0.00)	(0.00)
*Heavy work*	−0.01	−0.01	−0.01	−0.01
	(0.01)	(0.02)	(0.02)	(0.02)
*Sport*	0.04 ***	0.04 *	0.04 *	0.04 *
	(0.02)	(0.02)	(0.02)	(0.02)
ln*Retail*	0.00	0.00	0.00	0.00
	(0.03)	(0.05)	(0.05)	(0.05)
*Internet*	−0.02	−0.02	−0.02	−0.02
	(0.01)	(0.02)	(0.02)	(0.02)
Constant term	−8.77 ***	−8.77 *	−8.77 *	−8.77 *
	(3.08)	(4.73)	(4.74)	(4.74)
Year FE	YES	YES	YES	YES
Household FE	YES	YES	YES	YES
County FE	NO	NO	YES	YES
Province FE	NO	NO	NO	YES
Cluster robust standard errors	None	Village	Village	Village
Observations	5262	5262	5262	5262

Notes: Standard errors in parentheses; * *p* < 0.10, ** *p* < 0.05, *** *p* < 0.01.

**Table 5:** Estimation results of the COVID-19 impact on fat intake.

Variables	FE Model 1 (1)	FE Model 2 (2)	FE Model 3 (3)	FE Model 4 (4)
*COVID*	−0.0165 ***	−0.0165 ***	−0.0165 ***	−0.0165 ***
	(0.0036)	(0.0036)	(0.0037)	(0.0037)
*lnPrice_ch*	0.09 ***	0.09 *	0.09 *	0.09 *
	(0.02)	(0.05)	(0.05)	(0.05)
*lnPrice_fat*	−0.76 ***	−0.76 ***	−0.76 ***	−0.76 ***
	(0.02)	(0.06)	(0.06)	(0.06)
*lnPrice_pt*	0.22 ***	0.22 **	0.22 **	0.22 **
	(0.03)	(0.09)	(0.09)	(0.09)
ln*Exp*	2.39 ***	2.39 ***	2.39 ***	2.39 ***
	(0.57)	(0.85)	(0.85)	(0.85)
(ln*Exp*)^2^	−0.11 ***	−0.11 **	−0.11 **	−0.11 **
	(0.03)	(0.04)	(0.04)	(0.04)
*Family size*	−0.04 ***	−0.04 **	−0.04 **	−0.04 **
	(0.01)	(0.02)	(0.02)	(0.02)
*Non-farm work*	−0.00	−0.00	−0.00	−0.00
	(0.00)	(0.00)	(0.00)	(0.00)
*Heavy work*	−0.00	−0.00	−0.00	−0.00
	(0.01)	(0.02)	(0.02)	(0.02)
*Sport*	0.04 ***	0.04 *	0.04 *	0.04 *
	(0.01)	(0.02)	(0.02)	(0.02)
ln*Retail*	0.02	0.02	0.02	0.02
	(0.03)	(0.05)	(0.05)	(0.05)
*Internet*	−0.00	−0.00	−0.00	−0.00
	(0.01)	(0.02)	(0.02)	(0.02)
Constant term	−7.87 ***	−7.87 *	−7.87 *	−7.87 *
	(2.95)	(4.37)	(4.38)	(4.38)
Year FE	YES	YES	YES	YES
Household FE	YES	YES	YES	YES
County FE	NO	NO	YES	YES
Province FE	NO	NO	NO	YES
Cluster robust standard errors	None	Village	Village	Village
Observations	5262	5262	5262	5262

Notes: Standard errors in parentheses; * *p* < 0.10, ** *p* < 0.05, *** *p* < 0.01.

**Table 6:** Estimation results of the COVID-19 impact on protein intake.

Variables	FE Model 1 (1)	FE Model 2 (2)	FE Model 3 (3)	FE Model 4 (4)
*COVID*	−0.0115 ***	−0.0115 ***	−0.0115 ***	−0.0115 ***
	(0.0036)	(0.0037)	(0.0037)	(0.0037)
*lnPrice_ch*	0.05 **	0.05	0.05	0.05
	(0.02)	(0.05)	(0.05)	(0.05)
*lnPrice_fat*	0.31 ***	0.31 ***	0.31 ***	0.31 ***
	(0.02)	(0.06)	(0.06)	(0.06)
*lnPrice_pt*	−0.69 ***	−0.69 ***	−0.69 ***	−0.69 ***
	(0.04)	(0.08)	(0.08)	(0.08)
ln*Exp*	2.35 ***	2.35 **	2.35 **	2.35 **
	(0.58)	(0.93)	(0.93)	(0.93)
(ln*Exp*)^2^	−0.11 ***	−0.11 **	−0.11 **	−0.11 **
	(0.03)	(0.05)	(0.05)	(0.05)
*Family size*	−0.04 ***	−0.04 **	−0.04 **	−0.04 **
	(0.01)	(0.02)	(0.02)	(0.02)
*Non-farm work*	−0.00	−0.00	−0.00	−0.00
	(0.00)	(0.00)	(0.00)	(0.00)
*Heavy work*	−0.00	−0.00	−0.00	−0.00
	(0.01)	(0.02)	(0.02)	(0.02)
*Sport*	0.02	0.02	0.02	0.02
	(0.01)	(0.03)	(0.03)	(0.03)
ln*Retail*	0.02	0.02	0.02	0.02
	(0.03)	(0.05)	(0.05)	(0.05)
*Internet*	0.00	0.00	0.00	0.00
	(0.01)	(0.02)	(0.02)	(0.02)
Constant term	−7.98 ***	−7.98 *	−7.98 *	−7.98 *
	(2.97)	(4.76)	(4.77)	(4.77)
Year FE	YES	YES	YES	YES
Household FE	YES	YES	YES	YES
County FE	NO	NO	YES	YES
Province FE	NO	NO	NO	YES
Cluster robust standard errors	None	Village	Village	Village
Observations	5262	5262	5262	5262

Notes: Standard errors in parentheses; * *p* < 0.10, ** *p* < 0.05, *** *p* < 0.01.

### 3.3. Robustness Test

First, we assessed the robustness of the estimation results using fixed effects models with various dimensions (Table 3, Table 4, Table 5 and Table 6). In Columns (1) and (2), we only controlled for time and province fixed effects, while the standard errors in Columns (1) were not clustered robust. Then, in Columns (3), we added the county fixed effect. The results showed that the coefficients of variables were similar in all columns. Moreover, the estimation results using a fixed effect model (Table A1) were similar to those in Table 3, Table 4, Table 5 and Table 6. Therefore, the estimated results are robust.

Additionally, we replace expenditures with income in Equation (2). Due to the endogeneity associated with income measurement error, an instrumental estimation of the fixed effect model is constructed using expenditure as an instrumental variable. The results in Table A2 are generally consistent with those in Table 3, Table 4, Table 5 and Table 6, supporting the robustness of our study.

### 3.4. Heterogeneity Effect across Income Strata

In addition to identifying the heterogeneity in pandemic impact across different income groups, we also examined how the pandemic impacted rural residents with different income levels. In this paper, the entire sample was divided into four categories based on the percentile of per capita income: low-, middle-low-, middle-high-, and high-income groups. Specifically, the low-income group consisted of households in the lowest 25% of income brackets, the middle-low-income group consisted of households ranging from 25% to 50% of income brackets, the middle-high-income group consisted of households ranging from 50% to 75% of income brackets, and the high-income bracket comprised the remainder. The estimation results for different income groups are shown in Table 7.

The estimation results show that the COVID-19 pandemic had a significant and negative effect on the dietary macronutrient intake in the low-income group at the 5% level of significance. An increase of 100 confirmed cases in a county resulted in a 2.58% (*p* < 0.05), 2.18% (*p* < 0.05), 2.92% (*p* < 0.05), and 2.28% (*p* < 0.05) decrease in per capita dietary energy, carbohydrate, fat, and protein intake of the low-income rural residents. Furthermore, the fat intake of high-income rural residents decreased by 1.24% (*p* < 0.05) for every 100 confirmed cases in a county.

**Table 7:** Estimation results of the COVID-19 impact on nutritional intake by income groups.

Variables	Low Income	Middle-Low Income	Middle-High Income	High Income
ln*Dietary_Energy*	−0.0258 **	−0.0110	−0.0011	−0.0096 *
	(0.0106)	(0.0093)	(0.0069)	(0.0054)
ln*Carbohydrate*	−0.0218 **	−0.0092	−0.0009	−0.0108 *
	(0.0106)	(0.0097)	(0.0067)	(0.0056)
ln*Fat*	−0.0292 **	−0.0118	−0.0007	−0.0124 **
	(0.0122)	(0.0107)	(0.0069)	(0.0052)
ln*Protein*	−0.0228 **	−0.0036	−0.0044	−0.0068
	(0.0115)	(0.0100)	(0.0076)	(0.0051)
Observations	976	910	912	1036

Notes: For full estimation results, see supplementary materials (Tables S1–S4); standard errors in parentheses; * *p* < 0.10, ** *p* < 0.05.

## 4. Discussion

To the best of our knowledge, this paper is one of the first studies to investigate the COVID-19 pandemic’s impact on the nutritional intake of China’s rural residents. In order to prevent the spread of the virus, governments throughout China implemented a range of lockdown policies, including traffic control, production shut down, and restrictions on movement [46,50]. On the one hand, these measures disrupted agricultural production and food chain supplies and increased the cost of food storage and transportation [67,68]. On the other hand, disruptions in the supply of agricultural products, restrictions on human movement, and suspension of transportation and passenger transport caused the agricultural and non-farm incomes of rural residents to be reduced [69]. Additionally, rural residents’ expected income decreased when faced with epidemic-induced uncertainty, and they were more likely to upsurge precautionary saving motives as a result [70]. As a result of these factors, there was a decrease in food availability, a decrease in farmers’ willingness to consume, and consequently, a decrease in dietary energy intake. This provides an explanation for the main findings of this paper.

Further, since the negative effects of the COVID-19 pandemic on the intake of macronutrients differed, it is likely that the structure of the intake of macronutrients was altered as a result of the pandemic. The main reason why the nutritional structure changed was the changing structure of foods consumed. Thus, there was a relatively small decline in the consumption of carbohydrate-rich cereals as residents maintained their basic dietary needs. Meanwhile, fat-rich foods such as pork were consumed less frequently. Note that in 2020, China was also affected by the African swine fever outbreak, which contributed to a significant rise in pork prices and, to some extent, to a reduction in meat consumption. Using a time fixed effect, the impact of the African swine fever epidemic was controlled for in this study and thus did not affect our conclusions.

The study also found that low-income groups suffered significant and negative consequences in dietary intake from the COVID-19 pandemic. Since low-income groups have strong budget constraints and a high Engel coefficient, it was difficult to adjust the consumption structure when affected by the COVID-19 pandemic. Consequently, low-income households were less interchangeable across consumption types and food types. Under the influence of the pandemic, they could only reduce demand. Moreover, the impacts of the COVID-19 pandemic on the fat intake of high-income groups were also significant. However, their overall proportion of dietary energy from fat reached 42.9% in 2020, exceeding the maximum recommended value (30%) of the *Dietary Guidelines for Chinese Residents* (2016). Thus, the COVID-19 pandemic might improve the healthy diet of China’s high-income rural residents.

Several important policy implications are derived from the study. First, since the COVID-19 pandemic could exacerbate undernutrition among rural residents, particularly those with lower incomes, it is the government’s responsibility to ensure that low-income rural residents have access to sufficient nutrition. Second, there needs to be attention given to nutritional balance and dietary balance in light of COVID-19. Third, it is important for the government to introduce supply-side policies to stabilize production, as well as provide policies to promote consumption and price stability to make the food system more resilient [71]. Finally, under the influence of the COVID-19, China’s food security should focus on macro policy while focusing more on resident groups, families, and individuals.

It should be noted that our study has several limitations. First, the SAVE data contained only data regarding food consumption at home, which, despite being processed using Equation (3), does not accurately reflect food consumption away from home. Second, because of the limitations of the SAVE data, COVID-19 can only be evaluated with regard to macronutrients, and its effect cannot be assessed on micronutrients such as vitamins and minerals. Third, studies have shown that farmers can increase production diversity in order to enrich dietary diversity [72], but this factor was not taken into consideration in this study. Accordingly, we suggest that future studies should concentrate on the effects of COVID-19 on food consumption away from home, micronutrients, and production diversity in rural China.

## 5. Conclusions

In summary, based on nationwide panel data and a fixed effects model, this paper provides insights into the nutritional intake of China’s rural residents during the COVID-19 pandemic in 2020. We found that the COVID-19 pandemic negatively impacted the intake of dietary energy, carbohydrates, fats, and proteins. Furthermore, there was heterogeneity in the nutritional intake among different income groups, and the dietary intake of the low-income group was significantly affected by the COVID-19 pandemic. Therefore, the government should assist low-income groups in accessing sufficient nutrition during the COVID-19 epidemic.

## Data Availability

The data presented in this study are available upon request from the corresponding author.

## Supplementary Materials

The following supporting information can be downloaded at: https://www.mdpi.com/article/10.3390/nu14132704/s1, Table S1: Estimation results of the COVID-19 impact on dietary energy intake by income groups. Table S2: Estimation results of the COVID-19 impact on carbohydrate intake by income groups. Table S3: Estimation results of the COVID-19 impact on fat intake by income groups. Table S4: Estimation results of the COVID-19 impact on protein intake by income groups.

## Appendix A

**Table A1:** Estimation results from the ordinary fixed effect model.

Dependent Variables	ln*Energy*	ln*Carbohydrate*	ln*Fat*	ln*Protein*
*COVID*	−0.0130 ***	−0.0142 ***	−0.0165 ***	−0.0115 ***
	(0.0036)	(0.0038)	(0.0036)	(0.0036)
*Year2020*	0.03 ***	0.03 ***	0.04 ***	0.02 ***
	(0.01)	(0.01)	(0.01)	(0.01)
ln*Price_energy*	−0.48 ***			
	(0.02)			
ln*Price_ch*		−0.87 ***	0.09 ***	0.05 **
		(0.02)	(0.02)	(0.02)
ln*Price_fat*		0.32 ***	−0.76 ***	0.31 ***
		(0.02)	(0.02)	(0.02)
ln*Price_pt*		0.16 ***	0.22 ***	−0.69 ***
		(0.04)	(0.03)	(0.04)
ln*Exp*	2.40 ***	2.53 ***	2.39 ***	2.35 ***
	(0.57)	(0.60)	(0.57)	(0.58)
(ln*Exp*)^2^	−0.11 ***	−0.11 ***	−0.11 ***	−0.11 ***
	(0.03)	(0.03)	(0.03)	(0.03)
*Family size*	−0.04 ***	−0.04 ***	−0.04 ***	−0.04 ***
	(0.01)	(0.01)	(0.01)	(0.01)
*Non-farm work*	−0.00	−0.00	−0.00	−0.00
	(0.00)	(0.00)	(0.00)	(0.00)
*Heavy work*	−0.01	−0.01	−0.00	−0.00
	(0.01)	(0.01)	(0.01)	(0.01)
*Sport*	0.04 **	0.04 ***	0.04 ***	0.02
	(0.01)	(0.02)	(0.01)	(0.01)
ln*Retail*	0.05 *	0.00	0.02	0.02
	(0.03)	(0.03)	(0.03)	(0.03)
*Internet*	−0.01	−0.02	−0.00	0.00
	(0.01)	(0.01)	(0.01)	(0.01)
Constant	−6.32 **	−8.78 ***	−7.89 ***	−7.99 ***
	(2.96)	(3.08)	(2.95)	(2.97)
Household FE	Yes	Yes	Yes	Yes
Observations	5262	5262	5262	5262

Notes: Standard errors in parentheses; * *p* < 0.10, ** *p* < 0.05, *** *p* < 0.01.

**Table A2:** Estimation results from the instrumental fixed effect model.

Dependent Variables	ln*Energy*	ln*Carbohydrate*	ln*Fat*	ln*Protein*
*COVID*	−0.0281 ***	−0.0296 ***	−0.0309 ***	−0.0239 ***
	(0.0096)	(0.0104)	(0.0097)	(0.0086)
*Year2020*	0.07 ***	0.07 ***	0.08 ***	0.05 ***
	(0.02)	(0.02)	(0.02)	(0.02)
ln*Price_energy*	−0.50 ***			
	(0.05)			
ln*Price_ch*		−0.78 ***	0.17 ***	0.12 **
		(0.06)	(0.06)	(0.05)
ln*Price_fat*		0.30 ***	−0.78 ***	0.29 ***
		(0.06)	(0.05)	(0.05)
ln*Price_pt*		0.01	0.09	−0.80 ***
		(0.10)	(0.10)	(0.09)
ln*Inc*	0.56 ***	0.62 ***	0.58 ***	0.49 ***
	(0.18)	(0.20)	(0.19)	(0.16)
*Family size*	0.07 *	0.08 *	0.07 *	0.06
	(0.04)	(0.04)	(0.04)	(0.04)
*Non-farm work*	−0.00 ***	−0.00 ***	−0.00 ***	−0.00 ***
	(0.00)	(0.00)	(0.00)	(0.00)
*Heavy work*	−0.21 ***	−0.23 ***	−0.21 ***	−0.18 ***
	(0.07)	(0.08)	(0.08)	(0.07)
*Sport*	−0.05	−0.05	−0.05	−0.05
	(0.04)	(0.05)	(0.05)	(0.04)
ln*Retail*	−0.02	−0.05	−0.03	−0.03
	(0.07)	(0.07)	(0.07)	(0.06)
*Internet*	−0.07 **	−0.08 **	−0.06 *	−0.05 *
	(0.03)	(0.04)	(0.04)	(0.03)
Household FE	Yes	Yes	Yes	Yes
Cragg-Donald Wald F Statistics	12.02	11.88	11.88	11.88
Observations	5262	5262	5262	5262

Notes: Standard errors in parentheses; The Stock–Yogo weak ID test critical value (15% maximal IV size) is 8.96; * *p* < 0.10, ** *p* < 0.05, *** *p* < 0.01.
