# Supplementary material for: Impacts of COVID-19 on Nutritional Intake in Rural China: Panel Data Evidence
Source: Nutrients. 2022 Jun 29;14(13):2704. doi: 10.3390/nu14132704 (PMC9268832; doi:10.3390/nu14132704)
Supplement: Supplementary file 1 [file nutrients-14-02704-s001.zip › nutrients-1780999-supplementary.pdf]

Supplementary Materials

Impacts of COVID-19 on Nutritional Intake in Rural China: Panel Data Evidence

Xinru Han [1,†], Yufei Guo [2,3,†], Ping Xue [1], Xiudong Wang [1] and Wenbo Zhu [4,*]

Table S1. Estimation results of the COVID-19 impact on dietary energy intake by income groups.

Variables	Low Income	Middle-Low Income	Middle-High Income	High Income
Covid	−0.0258**	−0.0110	−0.0011	−0.0096*
	(0.0106)	(0.0093)	(0.0069)	(0.0054)
lnPrice_energy	−0.47***	−0.50***	−0.35***	−0.39***
	(0.07)	(0.11)	(0.10)	(0.11)
lnExp	−0.33	2.67*	2.97	1.33
	(1.81)	(1.56)	(2.01)	(1.85)
lnExpsq	0.02	−0.12	−0.13	−0.05
	(0.09)	(0.08)	(0.10)	(0.09)
Family_size	−0.10***	−0.11***	−0.06	−0.04
	(0.03)	(0.03)	(0.06)	(0.03)
Aworkday	−0.00	−0.00	0.00	0.00
	(0.00)	(0.00)	(0.00)	(0.00)
Heavywork	0.03	0.04	0.06	0.00
	(0.05)	(0.04)	(0.04)	(0.03)
Sport	0.05	0.00	0.02	0.03
	(0.03)	(0.06)	(0.03)	(0.03)
ln*Retail*	0.03	0.08	0.30***	0.00
	(0.08)	(0.06)	(0.11)	(0.07)
Internet	−0.00	−0.09	−0.06	0.02
	(0.03)	(0.05)	(0.05)	(0.03)
Constant	8.07	−7.70	−10.57	−0.87
	(9.54)	(7.98)	(9.99)	(9.53)
Observations	976	910	912	1,036
R-squared	0.97	0.98	0.96	0.97
Year FE	YES	YES	YES	YES
Household FE	YES	YES	YES	YES
County FE	YES	YES	YES	YES
Province FE	YES	YES	YES	YES
Cluster	Village	Village	Village	Village

Notes: Standard errors in parentheses; * $p < 0.10$, ** $p < 0.05$, *** $p < 0.01$.

Table S2. Estimation results of the COVID-19 impact on carbohydrate intake by income groups.

Variables	Low Income	Middle-Low Income	Middle-High Income	High Income
Covid	−0.0218**	−0.0092	−0.0009	−0.0108*
	(0.0106)	(0.0097)	(0.0067)	(0.0056)
lnPrice_ch	−0.82***	−0.92***	−0.63***	−0.92***
	(0.10)	(0.08)	(0.13)	(0.11)
lnPrice_fat	0.23***	0.13	0.53***	0.26**
	(0.09)	(0.18)	(0.13)	(0.13)
lnPrice_pt	0.23*	0.25	−0.17	0.33
	(0.12)	(0.21)	(0.21)	(0.23)
lnExp	−0.36	3.00*	2.48	1.46
	(1.77)	(1.63)	(2.08)	(1.68)
lnExpsq	0.03	−0.13	−0.11	−0.06
	(0.09)	(0.08)	(0.10)	(0.08)
Family_size	−0.09***	−0.11***	−0.06	−0.05
	(0.03)	(0.04)	(0.06)	(0.04)
Aworkday	−0.00	−0.00	0.00	0.00
	(0.00)	(0.00)	(0.00)	(0.00)
Heavywork	0.03	0.05	0.07**	−0.01
	(0.04)	(0.04)	(0.03)	(0.03)
Sport	0.05	0.01	0.02	0.03
	(0.04)	(0.05)	(0.04)	(0.03)
ln*Retail*	−0.02	0.02	0.27**	−0.01
	(0.08)	(0.07)	(0.12)	(0.07)
Internet	−0.01	−0.10	−0.06	0.01
	(0.03)	(0.06)	(0.06)	(0.03)
Constant	6.43	−11.13	−10.02	−3.91
	(9.17)	(8.32)	(10.41)	(8.90)
Observations	976	910	912	1,036
R-squared	0.99	0.99	0.99	0.99
Year FE	YES	YES	YES	YES
Household FE	YES	YES	YES	YES
County FE	YES	YES	YES	YES
Province FE	YES	YES	YES	YES
Cluster	Village	Village	Village	Village

Notes: Standard errors in parentheses; * $p < 0.10$, ** $p < 0.05$, *** $p < 0.01$.

Table S3. Estimation results of the COVID-19 impact on fat intake by income groups.

Variables	Low Income	Middle-Low Income	Middle-High Income	High Income
Covid	−0.0292**	−0.0118	−0.0007	−0.0124**
	(0.0122)	(0.0107)	(0.0069)	(0.0052)
lnPrice_ch	0.15	0.06	0.26**	0.05
	(0.09)	(0.07)	(0.13)	(0.11)
lnPrice_fat	−0.86***	−0.94***	−0.57***	−0.80***
	(0.08)	(0.16)	(0.12)	(0.12)
lnPrice_pt	0.25**	0.29	−0.02	0.37
	(0.11)	(0.20)	(0.20)	(0.24)
lnExp	−0.52	3.09*	2.45	1.03
	(1.77)	(1.59)	(1.86)	(1.69)
lnExpsq	0.03	-0.14*	-0.11	-0.04
	(0.09)	(0.08)	(0.09)	(0.08)
Family_size	−0.09***	−0.11***	−0.07	−0.04
	(0.03)	(0.04)	(0.06)	(0.04)
Aworkday	−0.00	−0.00	0.00	0.00
	(0.00)	(0.00)	(0.00)	(0.00)
Heavywork	0.02	0.04	0.07**	−0.00
	(0.04)	(0.04)	(0.03)	(0.03)
Sport	0.05	0.01	0.02	0.02
	(0.04)	(0.06)	(0.03)	(0.03)
ln*Retail*	−0.01	0.05	0.29***	−0.01
	(0.09)	(0.07)	(0.11)	(0.07)
Internet	−0.00	−0.08	−0.03	0.02
	(0.03)	(0.06)	(0.05)	(0.03)
Constant	7.62	−11.42	−9.75	−1.43
	(9.24)	(8.19)	(9.36)	(8.93)
Observations	976	910	912	1,036
R-squared	0.97	0.98	0.96	0.98
Year FE	YES	YES	YES	YES
Household FE	YES	YES	YES	YES
County FE	YES	YES	YES	YES
Province FE	YES	YES	YES	YES
Cluster	Village	Village	Village	Village

Notes: Standard errors in parentheses; * $p < 0.10$, ** $p < 0.05$, *** $p < 0.01$.

Table S4. Estimation results of the COVID-19 impact on protein intake by income groups.

Variables	Low Income	Middle-Low Income	Middle-High Income	High Income
Covid	−0.0228**	−0.0036	−0.0044	−0.0068
	(0.0115)	(0.0100)	(0.0076)	(0.0051)
lnPrice_ch	0.12	0.02	0.22	0.01
	(0.10)	(0.07)	(0.14)	(0.11)
lnPrice_fat	0.20**	0.09	0.55***	0.29**
	(0.09)	(0.16)	(0.14)	(0.11)
lnPrice_pt	−0.67***	−0.57***	−0.96***	−0.59***
	(0.12)	(0.19)	(0.21)	(0.21)
lnExp	−0.55	2.66	3.44	1.01
	(1.79)	(1.63)	(2.14)	(1.69)
lnExpsq	0.04	−0.12	−0.16	−0.04
	(0.09)	(0.08)	(0.11)	(0.08)
Family_size	−0.10***	−0.11***	−0.09	−0.04
	(0.03)	(0.04)	(0.07)	(0.03)
Aworkday	−0.00	−0.00	0.00	0.00
	(0.00)	(0.00)	(0.00)	(0.00)
Heavywork	0.01	0.04	0.07*	−0.01
	(0.04)	(0.04)	(0.03)	(0.04)
Sport	0.04	−0.02	−0.02	−0.01
	(0.03)	(0.07)	(0.04)	(0.02)
ln*Retail*	−0.01	0.05	0.28**	0.01
	(0.08)	(0.07)	(0.12)	(0.07)
Internet	−0.01	−0.09	−0.02	0.02
	(0.03)	(0.06)	(0.05)	(0.03)
Constant	7.18	−9.42	−14.90	−1.48
	(9.28)	(8.38)	(10.69)	(8.94)
Observations	976	910	912	1,036
R-squared	0.98	0.98	0.97	0.98
Year FE	YES	YES	YES	YES
Household FE	YES	YES	YES	YES
County FE	YES	YES	YES	YES
Province FE	YES	YES	YES	YES
Cluster	Village	Village	Village	Village

Notes: Standard errors in parentheses; * $p < 0.10$, ** $p < 0.05$, *** $p < 0.01$.
